# A Role for *Mycobacterium tuberculosis* Sigma Factor C in Copper Nutritional Immunity

**DOI:** 10.3390/ijms22042118

**Published:** 2021-02-20

**Authors:** Benjamin T. Grosse-Siestrup, Tuhina Gupta, Shelly Helms, Samantha L. Tucker, Martin I. Voskuil, Frederick D. Quinn, Russell K. Karls

**Affiliations:** 1 Department of Infectious Diseases, College of Veterinary Medicine, University of Georgia, Athens, GA 30602, USA; benjamin.gs@outlook.com (B.T.G.-S.); tgupta@uga.edu (T.G.); shellym7@uga.edu (S.H.); sltucker@uga.edu (S.L.T.); fquinn@uga.edu (F.D.Q.); 2 Department of Immunology and Microbiology, University of Colorado Anschutz Medical Campus, Aurora, CO 80045, USA; martin.voskuil@cuanschutz.edu

**Keywords:** *Mycobacterium tuberculosis*, sigma factor C (SigC), copper, nutritional immunity, nonribosomal peptide synthase, PPE1

## Abstract

Sigma factor C (SigC) contributes to *Mycobacterium tuberculosis* virulence in various animal models, but the stress response coordinated by this transcription factor was undefined. The results presented here indicate that SigC prevents copper starvation. Whole genome expression studies demonstrate short-term (4-h) induction of *sigC*, controlled from a tetracycline-inducible promoter, upregulates *ctpB* and genes in the nonribosomal peptide synthase (*nrp*) operon. These genes are expressed at higher levels after 48-h *sigC* induction, but also elevated are genes encoding copper-responsive regulator RicR and RicR-regulated copper toxicity response operon genes *rv0846–rv0850*, suggesting prolonged *sigC* induction results in excessive copper uptake. No growth and global transcriptional differences are observed between a *sigC* null mutant relative to its parent strain in 7H9 medium. In a copper-deficient medium, however, growth of the *sigC* deletion strain lags the parent, and 40 genes (including those in the *nrp* operon) are differentially expressed. Copper supplementation reverses the growth defect and silences most transcriptional differences. Together, these data support SigC as a transcriptional regulator of copper acquisition when the metal is scarce. Attenuation of *sigC* mutants in severe combined immunodeficient mice is consistent with an inability to overcome innate host defenses that sequester copper ions to deprive invading microbes of this essential micronutrient.

## 1. Introduction

*Mycobacterium tuberculosis* is the primary cause of death in humans by a single bacterial pathogen. In 2019, an estimated 10 million people developed tuberculosis (TB) disease and 1.4 million individuals died from TB; 208,000 of the deaths occurred among persons co-infected with HIV [1]. Globally, latent tuberculosis infections have been estimated at 1.7 billion [2].

The success of *M. tuberculosis* as a pathogen is in part due to sensing stress cues in the host and responding through implementation of effective countermeasures. In *M. tuberculosis*, sigma factor C (SigC) is one of 13 transcription sigma factors [3,4]. Mutants lacking intact *sigC* are attenuated in murine and guinea pig infection models [5,6,7]; however, these null mutants failed to yield phenotypes in vitro such as slower intracellular replication in phagocytyic cells [5,7]. Transcription from two start sites mapped upstream of *sigC* is detected in a *sigC* null mutant suggesting that this gene is not transcriptionally autoregulated [8]. Elevated *sigC* expression was not detected in response to a variety of stress stimuli [9]. Although *sigC* is fully conserved in *M. tuberculosis* strains CDC1551 and H37Rv, and in *M. bovis* BCG, none of the genes defined as members of the SigC regulon by transcriptome analyses of a CDC1551 *sigC* mutant [7] were upregulated after 18-h induction of *sigC* controlled from a tetracycline-inducible promoter in strain H37Rv [10] nor were any found with upstream promoters bound by SigC-RNA polymerase in *M. bovis* BCG chromosome immunoprecipitation/DNA microarray studies [11]. In the present study, phenotypes and gene expression associated with *sigC* were examined in derivatives of *M. tuberculosis* strain Erdman.

Transition metals are essential for all living organisms. Iron and copper commonly function as cofactors of enzymes that catalyze electron transfer reactions [12]. The electrostatic properties of these trace elements can stabilize reaction intermediates, but toxicity can result if such metals are present in excess [13]. Acquisition of trace elements from a host is a requirement for pathogenic bacteria. The term nutritional immunity was first used to define host processes of iron sequestration to prevent the growth of invading microbes [14], but subsequently expanded to encompass host sequestration of any essential trace metal from potential pathogens (reviewed in [15]). From an antimicrobial perspective, levels of free copper ions are maintained at extremely low levels by complexing or chaperoning the metal in most eukaryotic systems, an exception being phagosomes where copper ions are concentrated to target engulfed microbes [16,17]. The *M. tuberculosis* cytochrome *aa3–bc1* supercomplex is a member of the heme-copper respiratory oxidase family. All saturation transposon mutagenesis studies indicate *ctaD* (*rv3043*), encoding cytochrome *aa3* subunit 1 that contains the copper-B catalytic site, is an essential gene in *M. tuberculosis* [18,19,20]. As an obligate aerobe [21], *M. tuberculosis* must acquire copper to replicate. The data presented herein support SigC as a transcription factor required by the obligate human pathogen *M. tuberculosis* to acquire copper from environments where levels of the free metal are very low. 

## 2. Results and Discussion 

### 2.1. Absence of Significant Growth or Global Gene Expression Differences after Deletion of sigC from M. tuberculosis Strain Erdman Cultured in 7H9 Medium

**Figure 1:**
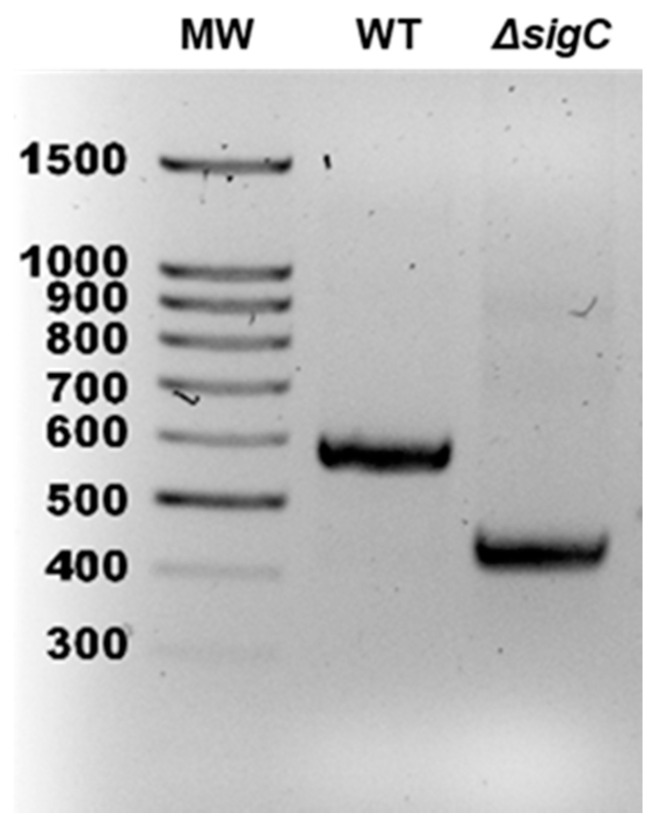
Confirmation of *Mycobacterium tuberculosis* Erdman Δ*sigC*. PCR analysis was used to confirm the 138-bp deletion within *sigC* of mutant (Δ*sigC*) relative to parent strain Erdman (WT). PCR bands of 587 and 449 bp are expected for WT and Δ*sigC*, respectively.

To aid in identifying *sigC*-associated phenotypes, an internal *sigC* deletion mutant (Δ*sigC*) was generated in *M. tuberculosis* strain Erdman (Figure 1). Since *sigC* is transcribed at high levels under many conditions [9], to minimize altering potential antisense effects on the downstream divergently transcribed *cobK* gene, an in-frame, internal portion of *sigC* was removed. The deletion removes bases encoding amino acids 123–168, which comprise most of sigma factor region 4 (amino acids 123–175) [22]. SigC has conserved sigma-70 family regions 2 and 4 for SigC-RNA polymerase binding to the -10 and -35 promoter regions, respectively, but lacks region 3 that aids promoter binding through contact with the extended -10 region [22]. Our previous studies showed reduced virulence in guinea pigs of an *M. tuberculosis* strain H37Rv derivative with the same *sigC* lesion, but the parent strain was more attenuated than expected [5]. We subsequently learned that this H37Rv strain was passaged on solid medium many years longer than standard freezer-stocked H37Rv isolates. Thus, to minimize the likelihood that a SigC-regulated gene may have been mutated during long-term passage, we used strain Erdman to generate the new mutant. The Erdman Δ*sigC* strain exhibited growth kinetics in Middlebrook 7H9 broth indistinguishable from strain Erdman and the complemented *sigC* mutant (Supplementary materials Figure S1). This was anticipated as *sigC* mutants of CDC1551 and H37Rv had no growth defects in 7H9 medium [5]. Transcriptomic studies using DNA microarrays with probes to the open reading frames in H37Rv examined global gene expression differences between Δ*sigC* and parent Erdman cultured to OD_600_ = 1 in 7H9 medium. Data obtained from four biological replicates hybridized on a total of ten slides revealed that no genes had significant differential expression of twofold or more after the data were analyzed using Significance Analysis of Microarrays (SAM) software (Table S1). The absence of significant gene expression differences was unexpected based on microarray results reported for a CDC1551Δ*sigC* mutant cultured in 7H9 medium, which identified over a dozen different genes repressed >2-fold in the mutant at OD_600_ = 0.5, OD_600_ = 2.0, and in stationary phase, but only *sigC* was downregulated in all growth conditions [7]. That study reported that two biological replicates were tested per strain per condition, which may underlie the inconsistent gene expression differences. The experiments to follow support why SigC function may not be required in 7H9 medium. 

### 2.2. Artificial sigC Induction Points to a Role for SigC in Metal Transport

**Figure 2:**
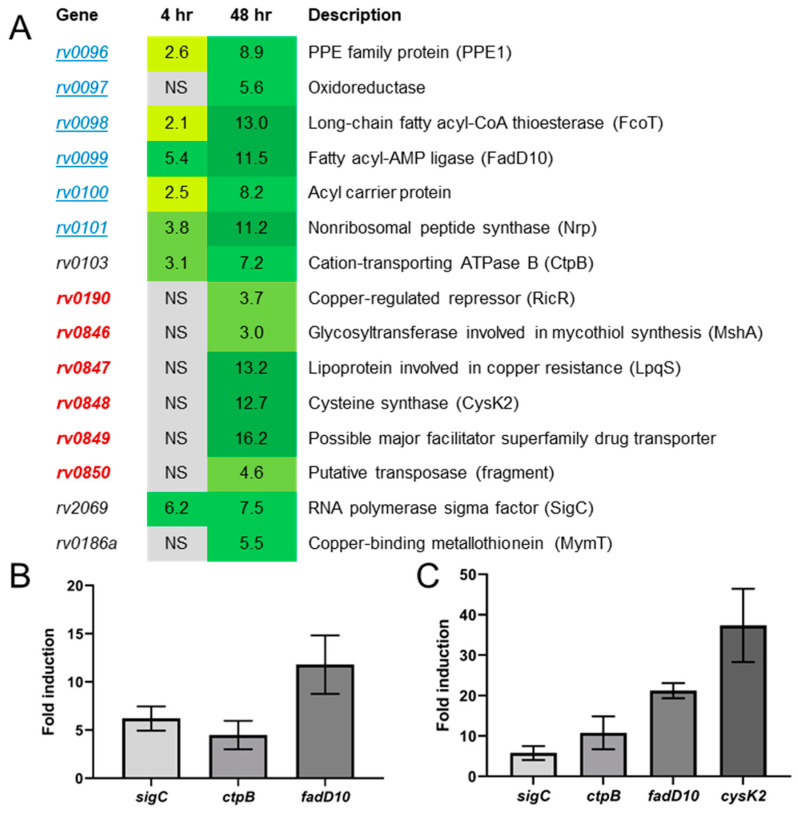
(**A**–**C**). Genes expressed >2-fold after 4- or 48-h *sigC* induction in Erdman in 7H9 medium. (**A**) Global gene expression studies using DNA microarrays in strain Erdman carrying a plasmid encoding *sigC* under control of a tetracycline-inducible promoter were performed in cultures grown to OD_600_ = 0.4 in 7H9tgADS prior to 4- or 48-h incubation with and without 50 ng/mL anhydrotetracycline induction. Genes with 2-fold or greater differential expression determined to be significant using Statistical Analysis for Microarrays software are shown. Genes in the *ppe1–nrp* operon are indicated (underlined, blue font). No genes were significantly downregulated more than 2-fold following artificial *sigC* induction. Genes regulated by RicR are shown (emboldened, red font). NS—not significant. Confirmation of microarray results by qRT-PCR of RNA from 4-h (**B**) and 48-h (**C**) *sigC* induction experiments. Results for *rv2069* (*sigC*), *rv0103* (*ctpB*), *rv0099* (*fadD10*), and *rv0848* (*cysK2*) were normalized to housekeeping gene *sigA*. Error bars indicate the standard deviation from the mean from a minimum of 3 biological replicates.

As significant gene expression differences were not detected between Δ*sigC* and strain Erdman in 7H9 cultures, transcriptome studies were performed with strain Erdman transformed with plasmid pSR173 encoding SigC with an N-terminal myc tag transcriptionally controlled from a tetracycline-inducible promoter. DNA microarray studies compared transcription of uninduced and anhydrotetracycline (aTc)-induced cells. After 4-h aTc induction, seven genes were significantly upregulated at least twofold (Figure 2A, column 2, Table S2). The artificially induced *sigC* gene was elevated 6.2-fold. The other genes with increased transcription were *ctpB* (*rv0103*), elevated 3.1-fold, and five of the six genes in the nearby *ppe1* (*rv0096*)–*nrp* (*rv0101*) operon, increased 2.1- to 5.4-fold. The microarray results were confirmed by qRT-PCR of *sigC*, *ctpB*, and *fadD10* (*rv0099*) only (Figure 2B). The *ctpB* gene encodes a cytoplasmic membrane P_1B_-type ATPase associated with transport of copper from mycobacterial cells [23]. Orthologs of genes in the *ppe1–nrp* operon are present in slow-growing pathogenic mycobacteria and in actinomyces [24]. The *rv0097–nrp* orthologous region from *Mycobacterium marinum* expressed in *Escherichia coli* enables synthesis of isonitrile lipopeptides when 2-decanoic or 2-dodecanoic acid is present in the cultures [24]. An isonitrile product from *Streptomyces thioluteus* binds copper and functions as a chalkophore for copper acquisition [25], while an *M. marinum* mutant lacking the *nrp* region was reported to accumulate less intracellular zinc [24].

To determine if the transcriptional profile changes after prolonged induction of *sigC*, expression after 48-h growth was examined. Consistent with the 4-h results, *sigC*, *ctpB*, and *ppe1–nrp* operon genes were upregulated, but to greater extents, ranging from 5.6- to 11.5-fold, (Figure 2A, Column 3, Table S3). Seven additional genes were significantly upregulated in response to 48-h *sigC* induction in Erdman (Figure 2A, Table S3). The regulated-in-copper repressor gene *ricR* was elevated (3.7-fold) as were five genes in the RicR-controlled *rv0846–rv0850* genomic region (ranging from 3.0- to 16.2-fold, Figure 2A). This region encodes a multi-copper oxidase (*rv0846*), a lipoprotein LpqS (*rv0847*), cysteine synthase CysK2 (*rv0848*), and a membrane permease (*rv0849*) that reduce the toxic effects of elevated intracellular copper levels [26]. Gene *rv0186a* encoding the copper-binding metallothionein MymT was increased 5.5-fold (Figure 2A). MymT protein binds up to six Cu(I) ions and *mymT* partially protects *M. tuberculosis* from copper toxicity [27]. Quantitative RT-PCR of *sigC*, *ctpB*, *rv0099*, and *rv0846* confirmed elevated transcription after 48-h *sigC* induction (Figure 2C). The 48-h *sigC* induction results are very similar to findings from a transcriptome study in which *sigC* was artificially induced for 18 h in an H37Rv background from a tetracycline-inducible promoter [10]. In that report, genes increased more than 2-fold included all six genes in the *ppe1–nrp* operon (4.2- to 5.9-fold), all five genes in the *rv0846–rv0850* region (2.6- to 3.7-fold), and *ctpB* (2.2-fold). The only other gene elevated after 18-h *sigC* induction was *rv1813* (2.0-fold) [10]; this gene was not increased in our studies. Finally, the *mymT* gene that we detected elevated after 48-h *sigC* induction was the only gene not upregulated after 18-h *sigC* induction [10]. This fits with the concept that bacteria accumulate more copper the longer *sigC* is artificially induced. Consistent with our 4- and 48-h *sigC*-induction data, Rustad and colleagues also did not detect any genes downregulated more than 2-fold after inducing *sigC* for 18 h.

**Figure 3:**
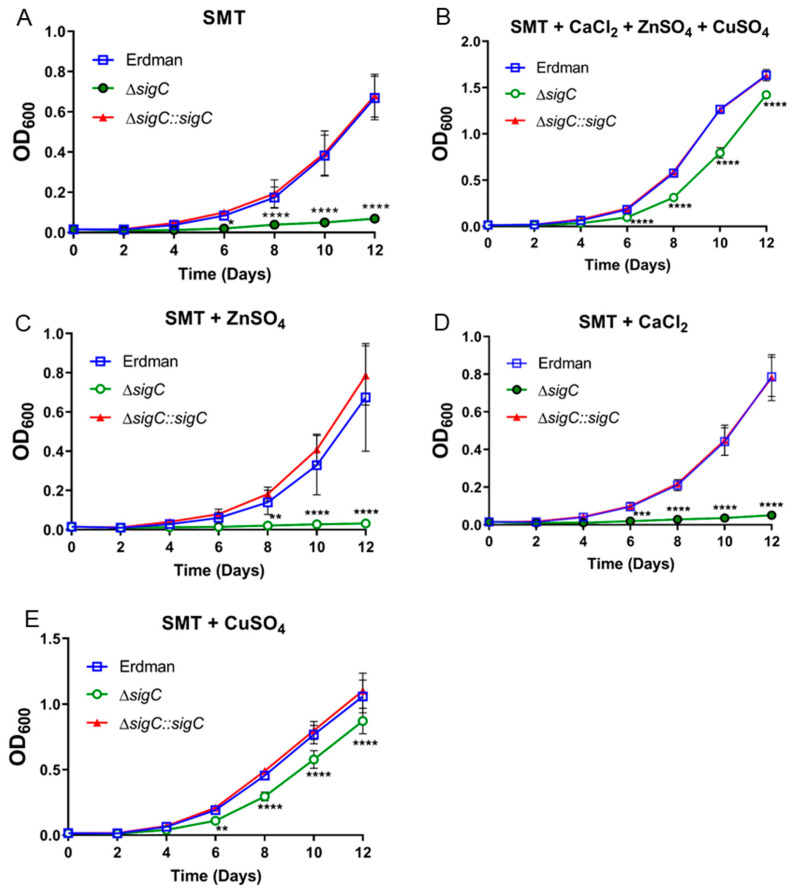
(**A**–**E**). Examination of metals on Δ*sigC* growth. Strains Erdman, Δ*sigC*, and the complemented *sigC* mutant (Δ*sigC::sigC*) were cultured in Sauton Medium with 0.025% Tyloxapol (SMT) alone (**A**) and after addition of one or more copper (6 µM), zinc (6 µM), or calcium (4.5 µM) salts (**B**–**E**). Culture densities shown are the average of two experiments with six independent replicates. One-way ANOVA was used to evaluate statistical significance (* *p* < 0.05, ** *p* < 0.01, *** *p* < 0.001, **** *p* < 0.0001).

Transcription of *ctpB* and the *ppe1–nrp* operon by SigC is consistent with results from in vivo chromosome immunoprecipitation/DNA microarray studies performed in *M. bovis* BCG that identified myc-SigC binding hotspots and almost identical predicted SigC-35 and -10 promoter elements immediately upstream of both regions [11]; note that the promoter region shown in Figure 3, panel C of that publication is mislabeled as *rv0095c*, instead of *rv0096* (*ppe1*). That study also demonstrated in vitro transcription of *ctpB* by RNA polymerase reconstituted with SigC, but did not report myc-SigC binding upstream of homologs of *ricR* or RicR-regulated genes. This suggests that *ctpB* and the *ppe1–nrp* operon are directly transcribed by Sig-RNA polymerase, but the copper toxicity-response genes are likely indirectly upregulated after prolonged artificial induction of *sigC* and actions of the encoded products of the *ppe1–nrp* operon and/or *ctpB*. Taken together, these results suggest that SigC functions in metal acquisition.

### 2.3. Identification of an In Vitro Phenotype for *Δ*sigC

To test the hypothesis that SigC functions in metal uptake, growth of the *sigC* mutant, *sigC*-complemented mutant, and strain Erdman was examined in a defined medium lacking copper, zinc, and calcium. To minimize nutrient carryover, cells initially cultured in 7H9 broth were washed with and subcultured into Sauton medium containing 0.025% of dispersant tyloxapol (SMT). After 3–4 passages in SMT medium, a phenotype developed in which growth of Δ*sigC* lagged the parent strain and complemented mutant (Figure 3A). To determine if the phenotype was due to lack of a specific metal present in 7H9 medium but lacking in SMT, growth was examined in SMT supplemented with the concentrations of copper, zinc, or calcium salts indicated in the formula for Middlebrook 7H9 medium in the BD Difco and BBL Manual [28]. Growth of Δ*sigC* was almost fully restored to wild type levels by supplementation with all three metals or with 6 µM copper sulfate alone, but not with either zinc or calcium salts (Figure 3B–E). These data confirm that SigC functions in copper acquisition.

### 2.4. Differential Gene Expression between Erdman and *Δ*sigC in SMT Supports SigC Function in Copper Uptake

**Figure 4:**
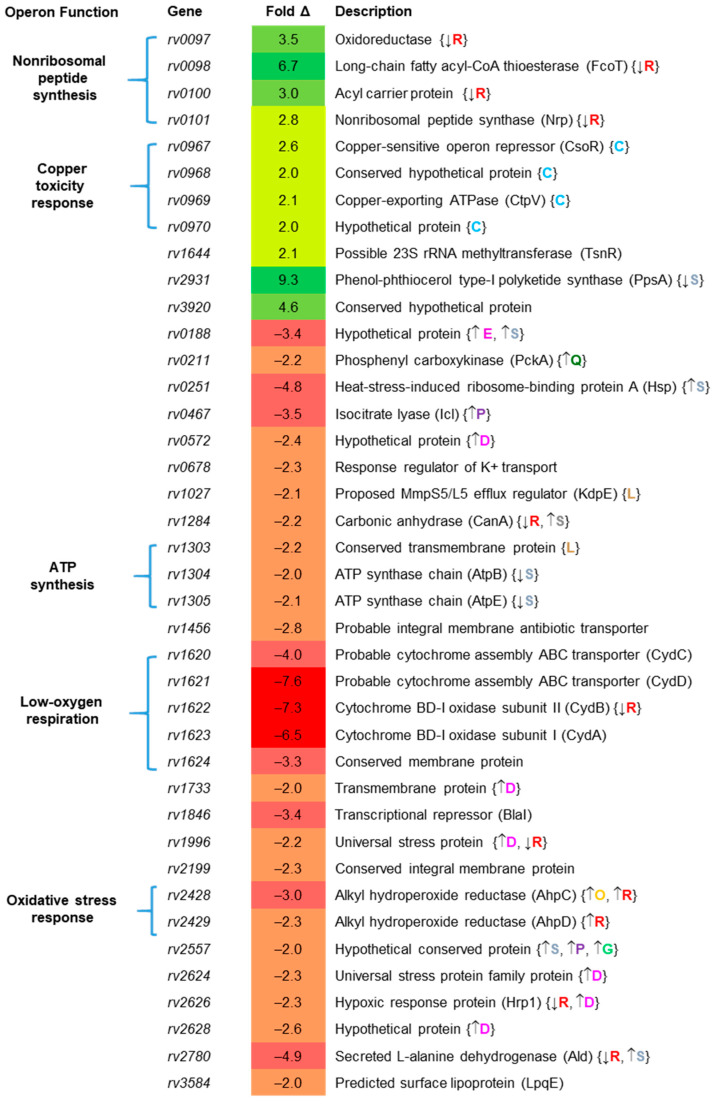
Genes differentially expressed >2-fold in Erdman and Δ*sigC* cultured in SMT. Genes predicted to be in the same operon are grouped by proposed operon function. Some of the identified conditions/regulators and direction (arrows) of specific gene are indicated (within {braces}). Code: C = copper/CsoR (light blue font) [31], D = DosR/hypoxia (pink font) [33], E = enduring hypoxic response (dark blue font) [34], G = in granulomas (green font) [35], L = RelMtb stringent response (brown font) [36], O = oxidative stress (orange font) [37], P = anaerobic persistence (purple font) [38], Q = acid and hypoxia (dark green font) [39], R = SenX3/RegX3 (red font) [29], S = in vitro starvation (gray font) [40]. Fold-changes for the genes shown are the average of 4 independent experiments. If fold-change was less than one, the (negative) reciprocal is listed. Genes with >2-fold significant differential expression after SAM analysis are shown.

To investigate how the growth defect of the *sigC* mutant in SMT lacking copper impacts gene expression, global transcriptional analysis between Δ*sigC* and parent Erdman was examined in SMT cultures grown to log phase (OD_600_ = 1). Cells were cultured as described for the growth studies (Section 2.3). Significant differential expression of twofold or greater was observed for 40 genes (Figure 4, Figure S2, Table S4). Of the 11 genes expressed at higher levels in Erdman, four were in the *ppe1–nrp* operon upregulated after artificial *sigC* induction in 7H9 medium (Figure 2). This operon is also upregulated in an *M. tuberculosis* mutant with a deletion in the *rv0490–rv0491* locus encoding the SenX3–RegX3 two-component regulator [29] which senses and responds to low phosphate levels [30]. In a Δ*sigC* mutant of *M. tuberculosis* strain CDC1551, expression of the *rv0490* homolog *mt0509* was reported to be downregulated in stationary phase 7H9 cultures [7]. Together, these observations indicate links between *sigC*, metal transport, and phosphate availability.

Other genes more highly expressed in Erdman relative to Δ*sigC* included the *csoR–rv968–ctpV–rv970* operon (Figure 4, Figure S2, Table S4). This operon is controlled by the copper-sensitive operon repressor, CsoR, which is distinct from the RicR-controlled operon that was upregulated in 7H9 cultures after 48-h *sigC* induction (Figure 2A). The *csoR* operon may be upregulated as a homeostatic mechanism after internalization of sufficient copper ions to bind CsoR and de-repression of its operon leading to production of the copper-exporting P-type ATPase CtpV to prevent toxic levels of copper from accumulating in the cytoplasm [31]. The *rv2931*/*ppsA* gene was the most-highly upregulated (9.3-fold) in Erdman relative to Δ*sigC* (Figure 4). It encodes a phenol-phthiocerol polyketide synthase that functions in phthiocerol dimycocerosates (PDIM) biosynthesis [32]. The elevated expression of *ppsA* in Erdman cultured in SMT medium but not when *sigC* was artificially induced in 7H9 medium suggests an indirect effect of *sigC* on *ppsA* expression, which will be discussed later. Gene *rv3920*, encoding a conserved protein of unknown function, was also significantly upregulated (4.6-fold). The only other gene significantly upregulated in the parent strain was *rv1644*/*tnsR* (2.1-fold) encoding a predicted 23S rRNA methyltransferase. It is unclear why these genes are elevated in a strain wild type for *sigC* under copper-limiting conditions. Of note, *ctpB* expression was not significantly upregulated in Erdman versus Δ*sigC* cultured in SMT despite *ctpB* being upregulated after 4- or 48-h *sigC* induction in 7H9 medium (Figure 2).

Among the 29 genes more highly expressed in Δ*sigC* relative to Erdman in SMT were operons that function in production of cytochrome *bd* oxidase (*rv1620–rv1624*), ATP synthesis (*rv1303–rv1305*), and alkyl hydroperoxide reductase (*rv2428–rv2429*) (Figure 4). Other genes upregulated in the mutant include several involved in central carbon metabolism, such as *icl* (encoding isocitrate lyase), *pckA* (encoding phosphoenol carboxykinase), *ald* (encoding alanine/glycine dehydrogenase), and *canA* (encoding carbonic anhydrase) (Figure 4). Elevated transcription of *cydB* and *icl* in the mutant was confirmed by qRT-PCR (Figure S2).

Genes upregulated in the *sigC* mutant in SMT cultures indicate alterations in central carbon metabolism and bioenergetics to alleviate reductive stress. Reductive stress results from the abnormal increase in the reduced forms of redox carriers such as NADH, NADPH, and FADH_2_ [41]. For detailed descriptions of *M. tuberculosis* central carbon metabolism and respiration, a number of reviews are recommended [42,43,44,45,46]. Under growth conditions with adequate aeration, the *M. tuberculosis* TCA cycle generates CO_2_ as well as NADH and FADH_2_. These carrier molecules are reoxidized by transfer of electrons through the menaquinone pool and the Qcr–Cta cytochrome *aa_3_–bc_1_* supercomplex to oxygen with concomitant pumping of protons across the cytoplasmic membrane to provide the proton motive force to drive ATP synthesis ([46], Figure S3). If the TCA cycle or electron transport chain is disrupted, then the reductive stress resulting from the buildup of reduced TCA cycle intermediates could lead to induction of metabolic processes in an effort to restore redox balance [41]. This appears to be the case for the *sigC* mutant (Figure S3). Increased *icl* expression allows the glyoxylate shunt to bypass the NADH- and CO_2_-generating steps in the TCA cycle that function in the production of succinate from α-ketoglutarate. Upregulation of *ald* enables production of amino acids glycine and alanine with concomitant oxidation of NADH to NAD. Increased transcription of *pckA* supplies phosphoenolpyruvate carboxykinase required for gluconeogenesis [47]. Expression of *pckA* is elevated by mildly acidic and hypoxic conditions [39]. The carbonic anhydrase encoded by *rv1284* is sensitive to oxidizing conditions [48]; thus, *rv1284* upregulation suggests a reduced redox state in which carbon fixation could serve as an electron sink to help restore redox balance. Metabolic flux analyses in carbon-limited chemostat studies have demonstrated that *M. tuberculosis* and *M. bovis* BCG can fix CO_2_ and utilize glyoxylate shunt and anapleurotic reactions for oxidation of pyruvate and production of succinyl CoA [49]. Elevated transcription of *cydABCD* is clearly a response to reductive stress. This operon is induced under hypoxic conditions and encodes a cytochrome *bd* oxidase (Cyd) which has higher oxygen affinity than the Qcr–Cta supercomplex, but is bioenergetically inefficient [46]. Importantly, unlike the Qcr–Cta supercomplexes, which are heme-copper oxygen reductases essential for efficient *M. tuberculosis* aerobic respiration, Cyd complexes only have heme centers for electron transfer to oxygen [50]. Upregulated Cyd production in response to copper starvation by Δ*sigC* in SMT would provide some copper-independent respiration, albeit with much less proton pumping across the membrane needed for ATP synthesis than respiration through the Qcr–Cta supercomplex (Figure S3). Upregulation of ATP synthesis genes *rv1303–rv1305* in the mutant suggests energy generation is affected.

Genes upregulated in the *sigC* mutant may also indicate responses to copper deficiency through inducing expression of copper-independent enzymes to mitigate respiratory distress. Alkyl hydroperoxide reductase genes *ahpCD* (*rv2428* and *rv2429*) upregulated by oxidating stress [37] may be upregulated in Δ*sigC* to provide an alternate defense against reactive oxygen species if insufficient copper was available to cofactor the mycobacterial Cu-Zn superoxide dismutase or other copper-dependent reductases. Genes *rv0572*, *rv1733*, *rv1996*, *rv2624*, *hrp1*, and *rv2628* are also induced as part of the DosR hypoxic response regulon [33,51]. Gene *rv0188* is a member of the enduring hypoxic response [34], while *rv2557* and *rv0467/icl* are upregulated during anaerobic persistence [38], with *rv2557* also elevated in the transition zone of human necrotic lung granulomas [35]. Hypoxia is expected from prolonged lack of aerobic respiration from copper-starved Qcr–Cta supercomplexes in the *sigC* mutant as cytochrome *bd* oxidases require three times more oxygen than heme-copper supercomplexes to pump the same number of protons to generate an equivalent amount of ATP (Figure S3). 

Upregulation of transcriptional regulators and genes controlled by other regulators in Δ*sigC* may be efforts to bypass different copper-dependent processes. Multiple redox-sensing systems would be expected to be affected by reductive stress and ATP depletion. The encoded product of *rv0572* is a predicted regulator the MmpS5/L5 efflux system [52]. The *blaI* gene encoding the beta-lactamase repressor is part of the iron-regulated repressor (IdeR) regulon [53], while *rv1305* and *atpE* are predicted members of the BlaI regulon [54]. Genes *ahpC*, *ahpD*, *ald*, *cydB*, *hrpI*, *rv1996*, and *rv2626* are regulated by the phosphate-responsive SenX3/RegX3 regulatory system [55]. Genes *rv0188*, *rv1284*, *hsp*, *atpB*, *atpE*, *rv2557*, and *ald* were reported to be upregulated after 96-h starvation [40], while *kdpE* and *rv1303* are part of the RelA-regulated stringent response [36]. Taken together, the seemingly disparate sets of genes with elevated expression in Δ*sigC* in copper-deficient SMT medium is consistent with a model in which these bacteria are upregulating the production of enzymes and processes that do not require copper for function.

### 2.5. Copper Supplementation Eliminates Most Transcriptional Differences between *Δ*sigC and Erdman

**Figure 5:**
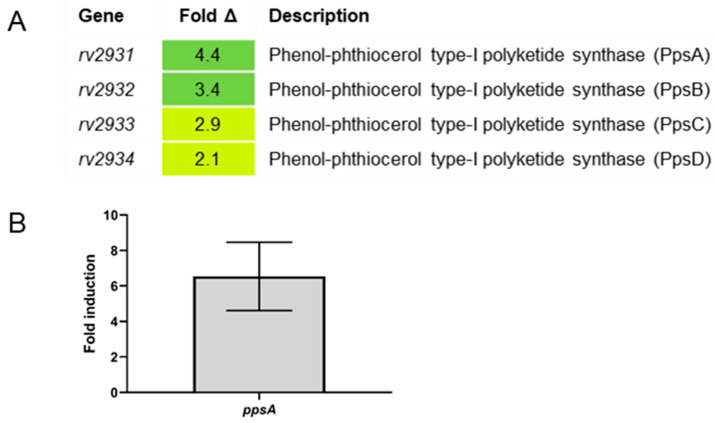
(**A**,**B**). Genes differentially expressed >2-fold in Erdman vs. Δ*sigC* cultured in copper-supplemented SMT medium. (**A**) Microarray studies were performed to examine global expression differences between strains Erdman and Δ*sigC* cultured to OD_600_ = 1 in SMT supplemented with 6 µM CuSO_4_. Microarrays used contained probes to the OFSs from *M. tuberculosis* strain H37Rv. Only genes with significant differential expression after SAM analysis of more than 2-fold are shown. (**B**) Expression of *rv2931* (*ppsA*) from strains cultured similarly was examined by qRT-PCR normalizing to *sigA*. Mean and standard error of 3 biological replicates is shown.

Based on the improved growth of Δ*sigC* in SMT supplemented with 6 μM copper sulfate (Figure 3E), we hypothesized that similar supplementation would eliminate many of the observed gene expression differences between Erdman and Δ*sigC* grown in SMT. This is precisely what we observed. Only 1 of the 40 genes that were differentially regulated twofold or more between Erdman and Δ*sigC* from SMT cultures (Figure 4) remained significantly elevated after 6 µM copper sulfate supplementation (Figure 5A, Table S5). 

The gene *ppsA* was again elevated in Erdman relative to Δ*sigC*, but also increased were genes *ppsB*, *ppsC*, and *ppsD*, located immediately downstream of *ppsA* and each encoding different phenol-phthiocerol polyketide synthases that function in PDIM lipid synthesis (Figure 5A). Elevated expression of this operon was confirmed by examination of *ppsA* by qRT-PCR (Figure 5B). It is unclear why PDIM synthesis operon genes were elevated in strain Erdman relative to the *sigC* mutant. No SigC promoters comparable to those preceding *ctpB* and *ppe1* were detected upstream of *ppsA*. The *sigC* mutant does not have a smooth colony morphology such as those observed for PDIM mutants, which indicates that *sigC* is not required for PDIM synthesis under all conditions. PDIM synthesis is upregulated in response to reductive stress by serving as an electron sink [56]. It is possible that mutations in PDIM synthesis develop in the *sigC* mutant upon passage in low-copper medium but eliminating a mechanism that mitigates reductive stress is unlikely to confer a growth advantage as Δ*sigC* depletes copper stores needed for respiration. Genes in the *pps* operon function in production of a common lipid core used for PDIM and glycosylated phenolphthiocerol dimycocerosate surface lipids [57]; thus, it is possible that under copper-starved conditions, the copper-chelating product of the *ppe1–nrp* operon enzymes is bound to the same common lipid core. The net effect would be a surface-exposed copper receptor for collecting trace amounts of copper from the environment. Reduced expression of *pps* genes in the *sigC* mutant in suboptimal copper conditions may reflect reduced need for the common lipid core due to an inability to produce the copper-chelating molecule. The transcriptional connection between *sigC* and PDIM genes may be indirect through redox-sensing factors. Transcription factor WhiB3 directly senses the redox state in *M. tuberculosis* through its thiol-disulfide redox switch and modulates the production of virulence lipids including PDIM [56]. PDIM synthesis is also impacted by one or more genes in the sigma factor M (SigM) operon. Deletion/replacement of H37Rv *sigM* with a kanamycin-resistance gene in the same orientation resulted in increased PDIM levels and expression of PDIM synthesis genes *ppsABCDE*, *ddrABC*, and *mas* [58]. As *sigM* is located upstream of genes encoding redox proteins thioredoxin and thioredoxin reductase, it is unclear if increased PDIM gene expression in the *sigM* mutant was due to loss of *sigM* or to altered transcription of downstream redox protein genes. It is also of note that artificial induction of *sigM* in the *sigM* mutant background resulted in 10-fold increased transcription of *ppe1–nrp* operon genes *rv0098–rv0100* [58]. Further studies are needed to understand the link between PDIM gene expression and *sigC* and potential interplay with other regulatory factors. Since 6 µM copper supplementation of SMT did not fully restore the growth rate of Δ*sigC* to that of parent strain Erdman (Figure 3E), a lower redox state in the *sigC* mutant due to a suboptimal level of copper-containing cytochrome *aa3–bc1* supercomplexes may continue to underlie the growth deficiency. If so, supplementing with a sufficient level of copper should fully restore growth of the mutant strain to equal that of its parent. 

### 2.6. Complete Reversal of the *Δ*sigC Growth Defect by 25 µM Copper Sulfate Addition

**Figure 6:**
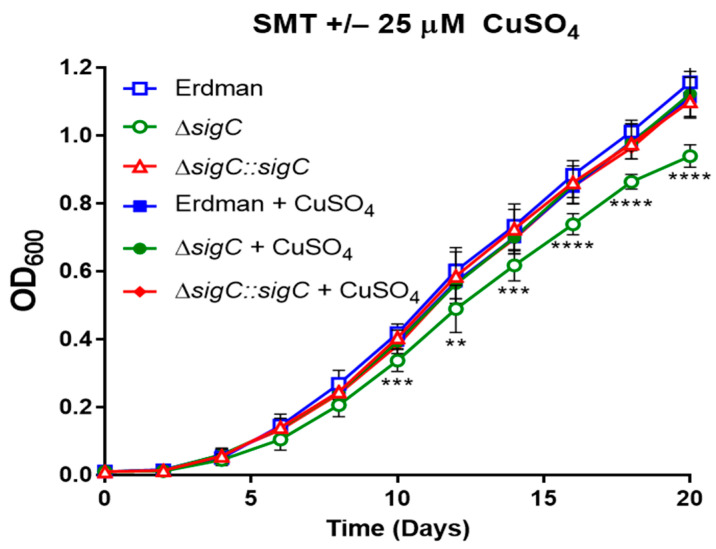
Restoration of wildtype growth of Δ*sigC* in SMT with 25 µM copper. Growth was monitored for strains Erdman, Δ*sigC*, and Δ*sigC::sigC* cultured in SMT medium without or with 25 µM copper sulfate supplementation. Results shown are the averages of 2 experiments with 3 replicates per strain. One-way ANOVA was used to assess statistical significance between groups (** *p* < 0.01, *** *p* < 0.001, **** *p* < 0.0001).

We assessed whether a higher concentration of copper greater than 6 µM would fully reverse the growth defect of Δ*sigC* to equal the replication rate of parent Erdman. We previously observed that growth of Δ*sigC* equals that of Erdman in 7H9 medium (Figure S1). While 6 µM copper sulfate is specifically added in the 7H9 broth base, additional copper is likely present associated with the post autoclave addition of bovine serum albumin. The average concentration of copper in healthy human serum is estimated at 17 µM [59]. We compared growth of Δ*sigC*, the complemented mutant, and Erdman in SMT in the presence or absence of 25 µM copper sulfate and observed that this level of supplementation fully restored the Δ*sigC* growth rate to equal those of wild type and the complemented mutant (Figure 6).

### 2.7. Attenuation of M. tuberculosis Erdman *Δ*sigC Virulence in SCID Mice 

**Figure 7:**
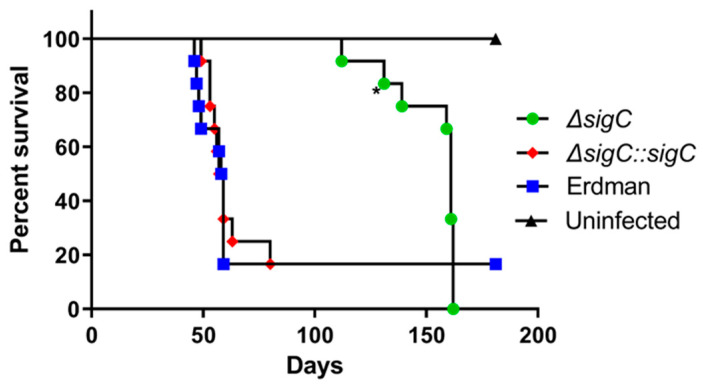
Survival of SCID mice infected with *M. tuberculosis* strains. Groups of 12 SCID mice were infected by intratracheal instillation with 0.025 mL of 10^4^ CFU/mL of strain Erdman, Δ*sigC*, or Δ*sigC::sigC*, or instilled with an equal volume of PBS. Animal survival differences between the *sigC* mutant and either the parent or complement group was analyzed by the Mantel–Cox test (* *p* < 0.05).

To determine if deletion of *sigC* from strain Erdman resulted in attenuation, groups of twelve SCID mice were infected in parallel by intratracheal instillation with 0.025 mL of 10^4^ CFU/mL of Δ*sigC*, Erdman, or the *sigC*-complemented mutant. Animals infected with Erdman or the complement had median survival times of 58 and 58.5 days, respectively (Figure 7). The Δ*sigC*-infected mice survived more than twice as long with a median survival of 161 days. Attenuation of Erdman Δ*sigC* in SCID mice is consistent with that reported for a CDC1551 Δ*sigC* strain wherein the mean survival time was 86 days following aerosol infection with 100 CFU of the mutant and only 26 days for animals infected with CDC1551 or the complemented CDC1551Δ*sigC* mutant [6]. The accelerated decline of animals aerosol-infected with parent strain CDC1551 relative to those infected with Erdman bacilli delivered intratracheally might be due to genetic differences between the strains or to more-efficient delivery of bacilli to the alveoli when bacterial suspensions are aerosolized. 

Since *sigC* is required for transcription of the *ppe1–nrp* operon under copper starvation conditions and *sigC* mutant strains are attenuated in animal models, mutants in the *ppe1–nrp* operon would also be expected to be highly attenuated. Studies with various mutant strains indicate this is the case. An *M. tuberculosis nrp* mutant was shown to be highly attenuated after infection in both immunocompetent (C57BL/6) and immunodeficient (SCID-, RAG2-, or IFNγ-deficient) murine models [60]. Mutant strains with disruption of the *M. bovis ppe1–nrp* operon by transposon insertion into the *rv0097* homolog resulted in a smooth colony phenotype, loss of PDIM synthesis, and avirulence upon infection of guinea pigs [61]. Transposon disruption of *rv0097* in *M. tuberculosis* reduced bacterial persistence in infected C57BL/6 mice, but surprisingly rendered the bacteria resistant to isoniazid in drug-treated mice, but not in axenic cultures [62]. Mutants of *M. tuberculosis* that fail to produce PDIMs display increased membrane permeability [63], reduced receptor-mediated macrophage infection [64], and reduced replication in murine lungs [65,66] and attenuated virulence [66]. It is unclear if all mutations that disrupt the *ppe1–nrp* operon directly result in loss of PDIM synthesis or if culture in copper-deficient conditions selects for suppressor mutations in PDIM synthesis genes to increase membrane permeability to more efficiently access copper. The Erdman Δ*sigC* stocks used for the animal studies were prepared in 7H9 medium prior to any studies involving growth in copper-deficient SMT. The CDC1551Δ*sigC* strain was also cultured in 7H9 medium [7]. As complementation of both mutants fully restored virulence, it is unlikely that spontaneous mutations blocking PDIM synthesis were responsible for the attenuation of *sigC* mutants in either *M. tuberculosis* background. 

The attenuation of *sigC* mutants in animal models and its essentiality in scavenging copper when the metal is scarce in vitro strongly supports its significance to an obligate pathogen like *M. tuberculosis* to overcome host copper nutritional immunity. The essentiality of the copper-requiring cytochrome *aa3–bc1* supercomplex underscores the importance of a copper-scavenging system to compete effectively for host copper. This is reflected in the conservation of the *ppe1–nrp* operon in slow-growing pathogenic mycobacteria, but not in fast-growing mycobacteria. The product(s) of the enzymes encoded in the *M. tuberculosis ppe1–nrp* operon remain to be elucidated. Although *rv0097–rv0100* substitute for the corresponding genes from *M. marinum* in isonitrile lipopeptide synthesis when co-expressed in *E. coli* with the *M. marinum nrp* ortholog, no products were detected with *M. tuberculosis rv0097–nrp* [24]. From the larger size of *M. tuberculosis nrp* (7539 bp) relative to the truncated *M. marinum* ortholog (4250 bp), the chemical structures of the final products are predicted to differ. While our data suggest that the *M. tuberculosis* product will function in copper acquisition, a role in the uptake of zinc or other transition metal has yet to be fully explored. 

### 2.8. Future Directions

A number of open questions remain in addition to defining the product of *M. tuberculosis nrp* operon enzymes. How does *M. tuberculosis* sense copper deficiency? The lack of differential expression of full-length *sigC* in Erdman versus *sigC* with an internal 138 bp deletion in Δ*sigC* suggests post transcriptional control of SigC. Activity of other sigma factors is often regulated by binding of an anti-sigma factor encoded in the same operon, but *sigC* is monocistronic and no anti-SigC factor has been identified to date. What is the relationship between control of *ppe1–nrp* operon genes by SigC and SigM? Why did overexpression of *sigM* in *M. tuberculosis* result in approximately ten-fold increased transcription of *rv0098–rv0100*, but not other genes in the operon [58], whereas *sigM* deletion had no effect on the same genes, but induced PDIM synthesis genes and production of the lipids [58]? Does SigM direct production of metal-binding isonitrile lipopeptides in response to metal starvation or other stress conditions or does it only function in the expression of lipid-transfer genes in the *ppe1–nrp* operon? What role does *ctpB* play in metal transport? Although *ctpB* is transcribed in vitro by SigC-RNA polymerase, the gene is not differentially expressed between a *sigC* mutant and its parent in copper-deficient medium. While this may suggest CtpB functions in copper export, it is also possible that the *sigC*-expressing bacteria had accumulated adequate amounts of copper prior to harvesting the cells for RNA analysis and elevated transcription was no longer necessary. These are active areas of investigation. 

## 3. Materials and Methods

### 3.1. Bacterial Strains and Culture Conditions

*Escherichia coli* strains were cultured in LB medium with 50 µg/mL kanamycin or 200 µg/mL hygromycin, as appropriate, for plasmid maintenance. *Mycobacterium tuberculosis* strain Erdman and derivatives were cultured at 37 °C with slow shaking (70 rpm) in Middlebrook 7H9 medium sterilized by autoclaving and then supplemented with 0.05% Tween 80, 0.5% glycerol, and 10% ADS (albumin, dextrose, NaCl) (7H9tgADS) [67] or in Sauton medium (0.05% KH_2_PO_4_, 0.05% MgSO_4_, 0.2% citric acid, 0.005% ferric ammonium citrate, 6% glycerol, 0.4% asparagine, pH adjusted to 7.4) with 0.025% Tyloxapol (SMT) added as a dispersant and sterilized by filtration. Water used for preparation of media and additives was obtained from a Type 1 ultrapure system (ELGA ULTRA GE MK2). Mycobacteria liquid culture vessels used were either (i) glass 125-mL sidearm flasks prepared by autoclaving for 90 min submerged in 1% Vesphene II solution, hand cleaned with a bristle brush, rinsed with Type 1 water, autoclaved for 90 minutes submerged in Type 1 water, rinsed with Type 1 water, and sterilized by autoclaving or (ii) new 250-mL wide-mouth polypropylene bottles (VWR) rinsed with Type 1 water and sterilized by autoclaving. The Δ*sigC* growth defect phenotype developed after 3–4 passages in SMT in either vessel type. For RNA isolation, bacteria were cultured in 7H9tgADS or SMT. For specific experiments, SMT was supplemented with the indicated concentrations of copper sulfate, zinc sulfate, and/or calcium chloride. Middlebrook 7H10 agar supplemented with 0.05% Tween 80, 0.5% glycerol, and 10% ADS (7H10tgADS) was used for plating mycobacteria. For *M. tuberculosis* strains harboring plasmids, 50 µg/mL hygromycin or 25 µg/mL kanamycin was used, as appropriate. 

A mutant with a 138-bp deletion within *sigC* was generated in *M. tuberculosis* strain Erdman by homologous recombination using methods previously described [5]. The Δ*sigC* mutant strain was confirmed by PCR of the *sigC* gene with primers 5′-GGT GGT TGC TCT TCC AAC ATG ACC GCG ACG GCA AGC-3′ and 5′-GGT GGT CTG CAG CTA GCC GGT GAG GTC GTC G-3′.

For complementation, plasmid pMV306sigC was constructed by PCR of *sigC* and ~600 bp of upstream DNA with primers 5′-GCT AAG CTT GCT CGT CCG TAG TCA C-3′ and 5′-CGC AAG CTT CGG TGG TCA TGA TAG C-3′ and inserted into the *Hin*dIII site of plasmid pMV306, which integrates into the *M. tuberculosis* chromosome at the mycobacteriophage L5 *attB* site [68]. After, pMV306sigC was electroporated into ∆*sigC* cultured in 7H9 medium and the plasmid was maintained with kanamycin selection.

For tetracycline-inducible *sigC* transcription, plasmid pSR173 which encodes SigC with an N-terminal myc tag and previously used in the myc-SigC chromosome immunoprecipitation/DNA microarray studies in *M. bovis* BCG [11] was kindly provided by Drs. Rodrigue and Gaudreau (University of Sherbrooke, Sherbrooke, QC, Canada) and maintained with hygromycin selection. 

### 3.2. RNA Isolation

For RNA isolation from *M. tuberculosis* cultures, cells were harvested by centrifugation (3500× *g*, 5 min) and cell pellets were frozen on dry ice and stored at −80 °C. Cells were thawed in the presence of Trizol (Gibco/Thermo Fisher Scientific, Waltham, MA, USA) and disrupted with 0.1 mm zirconium beads in a BeadBeater (Cole-Parmer, Vernon Hills, IL, USA) for three cycles of 40 s each at 4800 rpm with one minute cooling on ice between cycles. Next, chloroform was added and the tubes were mixed by inversion, incubated for three minutes, and the phases separated by centrifugation (12,000× *g*, 15 min). The aqueous phase was transferred to a new tube and extracted with an equal volume of acid phenol/chloroform. Following centrifugation (12,000× *g*, 10 min), the aqueous phase was transferred to a new tube and the RNA was isopropanol precipitated. Pelleted RNA was washed twice with 75% ethanol and suspended in diethyl pyrocarbonate-treated water. For DNA removal, each sample was treated twice with Turbo RNAse-free DNAse (Ambion/Thermo Fisher Scientific, Waltham, MA, USA) and RNA purified by RNeasy (Qiagen, Hilden, Germany) using the manufacturers’ instructions. After elution from RNeasy columns, 25 units RNaseOUT (Invitrogen/Thermo Fisher Scientific, Waltham, MA, USA) was added to each RNA sample. Residual genomic DNA contamination was assessed by PCR (40 cycles) with *sigA*-specific primers: 5′-AAC AGA TCG GCA AGG TAG-3′ and 5′AAC TTG TAC CCC TTG GTG-3′.

### 3.3. Microarray Analyses

The DNA microarray slides used in these studies were supplied by the TB Vaccine Testing and Research Materials Contract administered by Colorado State University, Fort Collins, CO (for *sigC* induction studies) and the M.I. Voskuil laboratory, University of Colorado Denver (for Erdman versus *sigC* mutant studies). All slides contained 70mer oligonucleotide probes for 4269 *M. tuberculosis* open reading frames (ORFs) from strains H37Rv and CDC with 26 controls. Complementary DNA (cDNA) was prepared using methods from the Pathogen Functional Genomics Resource Center at the J. Craig Venter Institute. Briefly, 2 µg RNA samples were incubated with 6 µg random hexamers (Invitrogen/Thermo Fisher Scientific, Waltham, MA, USA), 0.5 mM dNTP/aminoallyl-dUTPs, 2 µL SuperScript III Reverse Transcriptase, and 10 mM DTT overnight at 42 °C. The aminoallyl-labeled cDNAs produced were purified by MinElute PCR purification (Qiagen, Hilden, Germany) using manufacturer instructions except that the wash and elution buffers in the kit were replaced with phosphate wash buffer (5 mM KH_2_PO_4_, pH 8.0, 80% EtOH) and phosphate elution buffer (4 mM KH_2_PO_4_, pH 8.5), respectively. The cDNAs were next coupled to Cy3 or Cy5 dyes (Amersham Biosciences/Thermo Fisher Scientific, Waltham, MA, USA) and unbound dye was removed by MinElute PCR purification. The Cy3- and Cy5-labeled cDNAs were combined in pairs, dried, and suspended in hybridization buffer (5× SSC, 25% formamide, 0.1% SDS) with 0.4 mg/mL yeast tRNA, heated (98 °C, 2 min) and cooled briefly on ice. Microarray slides were prehybridized for a minimum of one hour at 42 °C by incubation in prehybridization buffer (5× SSC, 0.1% SDS, 1% BSA), washed stepwise with water and isopropanol, dried, and then hybridized with the labeled cDNA mixture 12–18 h at 42 °C. Slides were washed with high (0.1× SSC), medium (0.1× SSC, 0.1% SDS), and low (2× SSC, 0.1% SDS) stringency buffers, and fluorescence measured on a ProScanArray microarray scanner (Perkin Elmer, Waltham, MA, USA). Images collected were analyzed with ScanArrayExpress software and exported to Microsoft Excel. Each experiment consisted of a minimum of four biological replicates with at least two hybridizations performed per replicate. The dyes were swapped for the two hybridizations of each biological replicate. For each gene probe, eight intensity values or more were obtained. For normalization of the data, the LOWESS algorithm was used [69]. For identification of open reading frames (ORFs) with significant differential expression, the Significance Analysis of Microarrays (SAM) test procedure was applied using Excel SAM version 2.1 R package (http://statweb.stanford.edu/~tibs/SAM/Rdist/index.html (accessed on 24 December 2020)) set to a stringent false-discovery rate of zero. To ensure stringency, genes differentially regulated less than 2-fold have been excluded. 

### 3.4. Quantitative RT-PCR Assays

For cDNA generation, 500 ng RNA from each biological replicate was incubated with gene-specific primers and ImProm-II Reverse Transcriptase (Promega Corporation, Madison, WI, USA) following the manufacturer’s instructions. Quantitation of target transcripts was determined by PCR of 0.5 µL cDNA with Platinum SYBR Green Mastermix (Invitrogen/Thermo Fisher Scientific, Waltham, MA, USA) using a BioRad iCycler, and applying the ^∆∆^Ct method with normalization to *sigA* [70]. Three or more biological replicates were assayed for each gene. 

### 3.5. Animal Infection Studies

Procedures involving animals were conducted in accordance with protocols approved by the University of Georgia Institutional Animal Care and Use Committee (A2007-10135-0, approval date 28 February 2007) and in accordance with American Association for Accreditation of Laboratory Animal Care (AAALAS) policy. Severe-combined immunodeficient mice, female, 8–10 weeks of age, were purchased from Jackson Laboratories. All animals received ad libitum food and water. To assess the relative virulence of *M. tuberculosis* strains Δ*sigC*, Erdman, and the complemented *sigC* mutant, each strain was cultured in 7H9tgADS to OD_600_ = 1, counted microscopically, diluted in PBS + 0.05% Tween 80. Twelve mice per strain were infected by intratracheal instillation with 0.025 mL of 10^4^ CFU/mL stocks or sham-infected with 0.025 mL of phosphate-buffered saline (PBS). For survival studies, the mice were closely monitored for signs of disease or distress and humanely euthanized if moribund. The statistical significance of the Kaplan–Meier survival curve data was evaluated using the Mantel–Cox test within GraphPad Prism 9 software. 

### 3.6. Statistical Analysis

One-way ANOVA was used to assess statistical significance between groups for qRT-PCR studies (Figure 2B,C and Figure 6, Figure S2) and bacterial growth curves (Figure 3 and Figure 5, Figure S1). For animal survival studies, the Mantel–Cox test was employed. For microarray studies (Figure 2A, Figure 4, and Figure 6), the Statistical Analysis of Microarrays test (Excel SAM version 2.1 R package, Stanford University) was utilized to identify genes with significant differential expression. Numbers of sample replicates are indicated for each experiment. With the exception of the microarray studies, all statistical analyses were performed with GraphPad Prism ver. 9.0.0 (121) (GraphPad Software, Inc., San Diego, CA, USA).

## Data Availability

All data are contained within the article and supplementary materials.

## Supplementary Materials

Supplementary materials can be found at https://www.mdpi.com/1422-0067/22/4/2118/s1.

## References

[1] WHO (2020). Global Tuberculosis Report.

[2] Houben R.M., Dodd P.J. (2016). The Global Burden of Latent Tuberculosis Infection: A Re-estimation Using Mathematical Modelling. PLoS Med..

[3] Cole S.T., Brosch R., Parkhill J., Garnier T., Churcher C., Harris D., Gordon S.V., Eiglmeier K., Gas S., Barry C.E. (1998). Deciphering the biology of Mycobacterium tuberculosis from the complete genome sequence. Nature.

[4] Manganelli R. (2014). Sigma Factors: Key Molecules in Mycobacterium tuberculosis Physiology and Virulence. Microbiol. Spectr..

[5] Karls R.K., Guarner J., McMurray D.N., Birkness K.A., Quinn F.D. (2006). Examination of Mycobacterium tuberculosis sigma factor mutants using low-dose aerosol infection of guinea pigs suggests a role for SigC in pathogenesis. Microbiology.

[6] Abdul-Majid K.B., Ly L.H., Converse P.J., Geiman D.E., McMurray D.N., Bishai W.R. (2008). Altered cellular infiltration and cytokine levels during early Mycobacterium tuberculosis sigC mutant infection are associated with late-stage disease attenuation and milder immunopathology in mice. BMC Microbiol..

[7] Sun R., Converse P.J., Ko C., Tyagi S., Morrison N.E., Bishai W.R. (2004). Mycobacterium tuberculosis ECF sigma factor sigC is required for lethality in mice and for the conditional expression of a defined gene set. Mol. Microbiol..

[8] Chang A., Smollett K.L., Gopaul K.K., Chan B.H., Davis E.O. (2012). Mycobacterium tuberculosis H37Rv sigC is expressed from two promoters but is not auto-regulatory. Tuberculosis.

[9] Manganelli R., Dubnau E., Tyagi S., Kramer F.R., Smith I. (1999). Differential expression of 10 sigma factor genes in Mycobacterium tuberculosis. Mol. Microbiol..

[10] Rustad T.R., Minch K.J., Ma S., Winkler J.K., Hobbs S., Hickey M., Brabant W., Turkarslan S., Price N.D., Baliga N.S. (2014). Mapping and manipulating the Mycobacterium tuberculosis transcriptome using a transcription factor overexpression-derived regulatory network. Genome Biol..

[11] Rodrigue S., Brodeur J., Jacques P.E., Gervais A.L., Brzezinski R., Gaudreau L. (2007). Identification of mycobacterial sigma factor binding sites by chromatin immunoprecipitation assays. J. Bacteriol..

[12] Andreini C., Bertini I., Cavallaro G., Holliday G.L., Thornton J.M. (2008). Metal ions in biological catalysis: From enzyme databases to general principles. J. Biol. Inorg. Chem..

[13] Hood M.I., Skaar E.P. (2012). Nutritional immunity: Transition metals at the pathogen-host interface. Nat. Rev. Microbiol..

[14] Weinberg E.D. (1975). Nutritional immunity. Host’s attempt to withold iron from microbial invaders. JAMA.

[15] Hennigar S.R., McClung J.P. (2016). Nutritional Immunity: Starving Pathogens of Trace Minerals. Am. J. Lifestyle Med..

[16] Darwin K.H. (2015). Mycobacterium tuberculosis and Copper: A Newly Appreciated Defense against an Old Foe?. J. Biol. Chem..

[17] Fu Y., Chang F.M., Giedroc D.P. (2014). Copper transport and trafficking at the host-bacterial pathogen interface. Acc. Chem. Res..

[18] Sassetti C.M., Boyd D.H., Rubin E.J. (2003). Genes required for mycobacterial growth defined by high density mutagenesis. Mol. Microbiol..

[19] Griffin J.E., Gawronski J.D., Dejesus M.A., Ioerger T.R., Akerley B.J., Sassetti C.M. (2011). High-resolution phenotypic profiling defines genes essential for mycobacterial growth and cholesterol catabolism. PLoS Pathog..

[20] DeJesus M.A., Gerrick E.R., Xu W., Park S.W., Long J.E., Boutte C.C., Rubin E.J., Schnappinger D., Ehrt S., Fortune S.M. (2017). Comprehensive Essentiality Analysis of the Mycobacterium tuberculosis Genome via Saturating Transposon Mutagenesis. mBio.

[21] Koch A., Mizrahi V. (2018). Mycobacterium tuberculosis. Trends Microbiol..

[22] Rodrigue S., Provvedi R., Jacques P.E., Gaudreau L., Manganelli R. (2006). The sigma factors of Mycobacterium tuberculosis. FEMS Microbiol. Rev..

[23] Leon-Torres A., Arango E., Castillo E., Soto C.Y. (2020). CtpB is a plasma membrane copper (I) transporting P-type ATPase of Mycobacterium tuberculosis. Biol. Res..

[24] Harris N.C., Sato M., Herman N.A., Twigg F., Cai W., Liu J., Zhu X., Downey J., Khalaf R., Martin J. (2017). Biosynthesis of isonitrile lipopeptides by conserved nonribosomal peptide synthetase gene clusters in Actinobacteria. Proc. Natl. Acad. Sci. USA.

[25] Wang L., Zhu M., Zhang Q., Zhang X., Yang P., Liu Z., Deng Y., Zhu Y., Huang X., Han L. (2017). Diisonitrile Natural Product SF2768 Functions As a Chalkophore That Mediates Copper Acquisition in Streptomyces thioluteus. ACS Chem. Biol..

[26] Festa R.A., Jones M.B., Butler-Wu S., Sinsimer D., Gerads R., Bishai W.R., Peterson S.N., Darwin K.H. (2011). A novel copper-responsive regulon in Mycobacterium tuberculosis. Mol. Microbiol..

[27] Gold B., Deng H., Bryk R., Vargas D., Eliezer D., Roberts J., Jiang X., Nathan C. (2008). Identification of a copper-binding metallothionein in pathogenic mycobacteria. Nat. Chem. Biol..

[28] Zimbro M.J. (2009). Difco & BBL Manual: Manual of Microbiological Culture Media.

[29] Parish T., Smith D.A., Roberts G., Betts J., Stoker N.G. (2003). The senX3-regX3 two-component regulatory system of Mycobacterium tuberculosis is required for virulence. Microbiology.

[30] Rifat D., Belchis D.A., Karakousis P.C. (2014). senX3-independent contribution of regX3 to Mycobacterium tuberculosis virulence. BMC Microbiol..

[31] Marcus S.A., Sidiropoulos S.W., Steinberg H., Talaat A.M. (2016). CsoR Is Essential for Maintaining Copper Homeostasis in Mycobacterium tuberculosis. PLoS ONE.

[32] He W., Soll C.E., Chavadi S.S., Zhang G., Warren J.D., Quadri L.E. (2009). Cooperation between a coenzyme A-independent stand-alone initiation module and an iterative type I polyketide synthase during synthesis of mycobacterial phenolic glycolipids. J. Am. Chem. Soc..

[33] Park H.D., Guinn K.M., Harrell M.I., Liao R., Voskuil M.I., Tompa M., Schoolnik G.K., Sherman D.R. (2003). Rv3133c/dosR is a transcription factor that mediates the hypoxic response of Mycobacterium tuberculosis. Mol. Microbiol..

[34] Rustad T.R., Harrell M.I., Liao R., Sherman D.R. (2008). The enduring hypoxic response of Mycobacterium tuberculosis. PLoS ONE.

[35] Fenhalls G., Stevens L., Moses L., Bezuidenhout J., Betts J.C., Helden Pv P., Lukey P.T., Duncan K. (2002). In situ detection of Mycobacterium tuberculosis transcripts in human lung granulomas reveals differential gene expression in necrotic lesions. Infect. Immun..

[36] Dahl J.L., Kraus C.N., Boshoff H.I., Doan B., Foley K., Avarbock D., Kaplan G., Mizrahi V., Rubin H., Barry C.E. (2003). The role of RelMtb-mediated adaptation to stationary phase in long-term persistence of Mycobacterium tuberculosis in mice. Proc. Natl. Acad. Sci. USA.

[37] Sherman D.R., Mdluli K., Hickey M.J., Barry C.E., Stover C.K. (1999). AhpC, oxidative stress and drug resistance in Mycobacterium tuberculosis. Biofactors.

[38] Saxena A., Srivastava V., Srivastava R., Srivastava B.S. (2008). Identification of genes of Mycobacterium tuberculosis upregulated during anaerobic persistence by fluorescence and kanamycin resistance selection. Tuberculosis.

[39] Kim S.Y., Lee B.S., Shin S.J., Kim H.J., Park J.K. (2008). Differentially expressed genes in Mycobacterium tuberculosis H37Rv under mild acidic and hypoxic conditions. J. Med. Microbiol..

[40] Betts J.C., Lukey P.T., Robb L.C., McAdam R.A., Duncan K. (2002). Evaluation of a nutrient starvation model of Mycobacterium tuberculosis persistence by gene and protein expression profiling. Mol. Microbiol..

[41] Farhana A., Guidry L., Srivastava A., Singh A., Hondalus M.K., Steyn A.J.C., Poole R.K. (2010). Reductive Stress in Microbes: Implications for Understanding *Mycobacterium tuberculosis* Diseases and Persistence. Advances in Microbial Physiology.

[42] Rhee K.Y., de Carvalho L.P., Bryk R., Ehrt S., Marrero J., Park S.W., Schnappinger D., Venugopal A., Nathan C. (2011). Central carbon metabolism in Mycobacterium tuberculosis: An unexpected frontier. Trends Microbiol..

[43] Ehrt S., Rhee K. (2013). Mycobacterium tuberculosis metabolism and host interaction: Mysteries and paradoxes. Curr. Top. Microbiol. Immunol..

[44] Baughn A.D., Rhee K.Y. (2014). Metabolomics of Central Carbon Metabolism in Mycobacterium tuberculosis. Microbiol. Spectr..

[45] Ehrt S., Schnappinger D., Rhee K.Y. (2018). Metabolic principles of persistence and pathogenicity in Mycobacterium tuberculosis. Nat. Rev. Microbiol..

[46] Cook G.M., Hards K., Vilcheze C., Hartman T., Berney M. (2014). Energetics of Respiration and Oxidative Phosphorylation in Mycobacteria. Microbiol. Spectr..

[47] Marrero J., Rhee K.Y., Schnappinger D., Pethe K., Ehrt S. (2010). Gluconeogenic carbon flow of tricarboxylic acid cycle intermediates is critical for Mycobacterium tuberculosis to establish and maintain infection. Proc. Natl. Acad. Sci. USA.

[48] Nienaber L., Cave-Freeman E., Cross M., Mason L., Bailey U.M., Amani P., Davis R.A., Taylor P., Hofmann A. (2015). Chemical probing suggests redox-regulation of the carbonic anhydrase activity of mycobacterial Rv1284. FEBS J..

[49] Beste D.J., Bonde B., Hawkins N., Ward J.L., Beale M.H., Noack S., Noh K., Kruger N.J., Ratcliffe R.G., McFadden J. (2011). (1)(3)C metabolic flux analysis identifies an unusual route for pyruvate dissimilation in mycobacteria which requires isocitrate lyase and carbon dioxide fixation. PLoS Pathog..

[50] Giuffre A., Borisov V.B., Arese M., Sarti P., Forte E. (2014). Cytochrome bd oxidase and bacterial tolerance to oxidative and nitrosative stress. Biochim. Biophys. Acta.

[51] Voskuil M.I., Visconti K.C., Schoolnik G.K. (2004). Mycobacterium tuberculosis gene expression during adaptation to stationary phase and low-oxygen dormancy. Tuberculosis.

[52] Milano A., Pasca M.R., Provvedi R., Lucarelli A.P., Manina G., Ribeiro A.L., Manganelli R., Riccardi G. (2009). Azole resistance in Mycobacterium tuberculosis is mediated by the MmpS5-MmpL5 efflux system. Tuberculosis.

[53] Gold B., Rodriguez G.M., Marras S.A., Pentecost M., Smith I. (2001). The Mycobacterium tuberculosis IdeR is a dual functional regulator that controls transcription of genes involved in iron acquisition, iron storage and survival in macrophages. Mol. Microbiol..

[54] Sala C., Haouz A., Saul F.A., Miras I., Rosenkrands I., Alzari P.M., Cole S.T. (2009). Genome-wide regulon and crystal structure of BlaI (Rv1846c) from Mycobacterium tuberculosis. Mol. Microbiol..

[55] Roberts G., Vadrevu I.S., Madiraju M.V., Parish T. (2011). Control of CydB and GltA1 expression by the SenX3 RegX3 two component regulatory system of Mycobacterium tuberculosis. PLoS ONE.

[56] Singh A., Crossman D.K., Mai D., Guidry L., Voskuil M.I., Renfrow M.B., Steyn A.J. (2009). Mycobacterium tuberculosis WhiB3 maintains redox homeostasis by regulating virulence lipid anabolism to modulate macrophage response. PLoS Pathog..

[57] Simeone R., Constant P., Malaga W., Guilhot C., Daffe M., Chalut C. (2007). Molecular dissection of the biosynthetic relationship between phthiocerol and phthiodiolone dimycocerosates and their critical role in the virulence and permeability of Mycobacterium tuberculosis. FEBS J..

[58] Raman S., Puyang X., Cheng T.Y., Young D.C., Moody D.B., Husson R.N. (2006). Mycobacterium tuberculosis SigM positively regulates Esx secreted protein and nonribosomal peptide synthetase genes and down regulates virulence-associated surface lipid synthesis. J. Bacteriol..

[59] Li D.D., Zhang W., Wang Z.Y., Zhao P. (2017). Serum Copper, Zinc, and Iron Levels in Patients with Alzheimer’s Disease: A Meta-Analysis of Case-Control Studies. Front. Aging. Neurosci..

[60] Bhatt K., Machado H., Osorio N.S., Sousa J., Cardoso F., Magalhaes C., Chen B., Chen M., Kim J., Singh A. (2018). A Nonribosomal Peptide Synthase Gene Driving Virulence in Mycobacterium tuberculosis. mSphere.

[61] Hotter G.S., Wards B.J., Mouat P., Besra G.S., Gomes J., Singh M., Bassett S., Kawakami P., Wheeler P.R., de Lisle G.W. (2005). Transposon mutagenesis of Mb0100 at the ppe1-nrp locus in Mycobacterium bovis disrupts phthiocerol dimycocerosate (PDIM) and glycosylphenol-PDIM biosynthesis, producing an avirulent strain with vaccine properties at least equal to those of M. bovis BCG. J. Bacteriol..

[62] Dhar N., McKinney J.D. (2010). Mycobacterium tuberculosis persistence mutants identified by screening in isoniazid-treated mice. Proc. Natl. Acad. Sci. USA.

[63] Camacho L.R., Constant P., Raynaud C., Laneelle M.A., Triccas J.A., Gicquel B., Daffe M., Guilhot C. (2001). Analysis of the phthiocerol dimycocerosate locus of Mycobacterium tuberculosis. Evidence that this lipid is involved in the cell wall permeability barrier. J. Biol. Chem..

[64] Astarie-Dequeker C., Le Guyader L., Malaga W., Seaphanh F.K., Chalut C., Lopez A., Guilhot C. (2009). Phthiocerol dimycocerosates of M. tuberculosis participate in macrophage invasion by inducing changes in the organization of plasma membrane lipids. PLoS Pathog..

[65] Cox J.S., Chen B., McNeil M., Jacobs W.R. (1999). Complex lipid determines tissue-specific replication of Mycobacterium tuberculosis in mice. Nature.

[66] Kirksey M.A., Tischler A.D., Simeone R., Hisert K.B., Uplekar S., Guilhot C., McKinney J.D. (2011). Spontaneous phthiocerol dimycocerosate-deficient variants of Mycobacterium tuberculosis are susceptible to gamma interferon-mediated immunity. Infect. Immun..

[67] Braunstein M., Bardarov S.S., Jacobs W.R. (2002). Genetic methods for deciphering virulence determinants of Mycobacterium tuberculosis. Methods Enzymol..

[68] Kong D., Kunimoto D.Y. (1995). Secretion of human interleukin 2 by recombinant Mycobacterium bovis BCG. Infect. Immun..

[69] Yang Y.H., Dudoit S., Luu P., Lin D.M., Peng V., Ngai J., Speed T.P. (2002). Normalization for cDNA microarray data: A robust composite method addressing single and multiple slide systematic variation. Nucleic Acids Res..

[70] Livak K.J., Schmittgen T.D. (2001). Analysis of relative gene expression data using real-time quantitative PCR and the 2(-Delta Delta C(T)) Method. Methods.

[71] Grosse-Siestrup B.T. (2012). Examination of the SigC Regulon and Cobalamin Biosynthesis in Mycobacterium tuberculosis. Ph.D. Thesis.

