# Supplementary figures and images for: A Role for Mycobacterium tuberculosis Sigma Factor C in Copper Nutritional Immunity
Source: Int J Mol Sci. 2021 Feb 20;22(4):2118. doi: 10.3390/ijms22042118 (PMC7924339; doi:10.3390/ijms22042118)

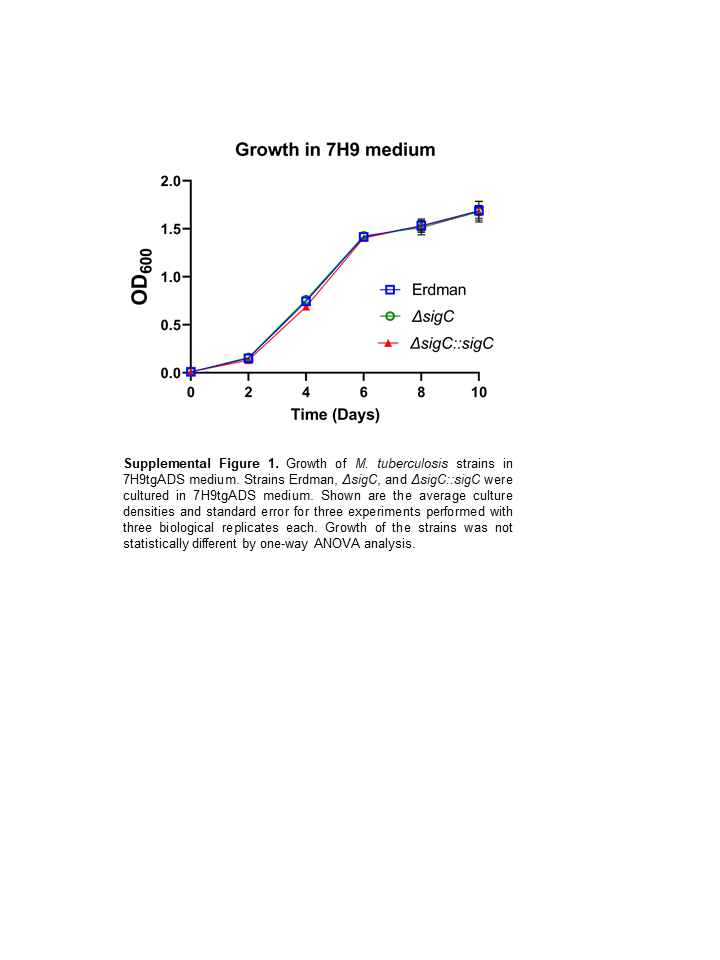

Supplement: Supplementary file 1 [file ijms-22-02118-s001.zip › Supplementary files/Figure S1.TIF]

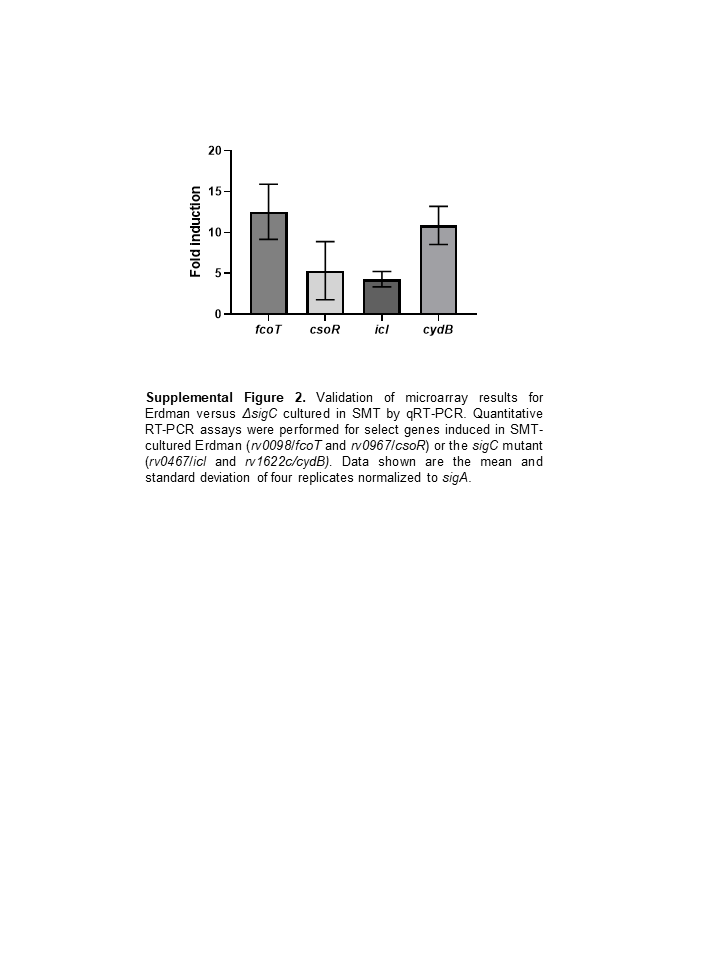

Supplement: Supplementary file 1 [file ijms-22-02118-s001.zip › Supplementary files/Figure S2.TIF]

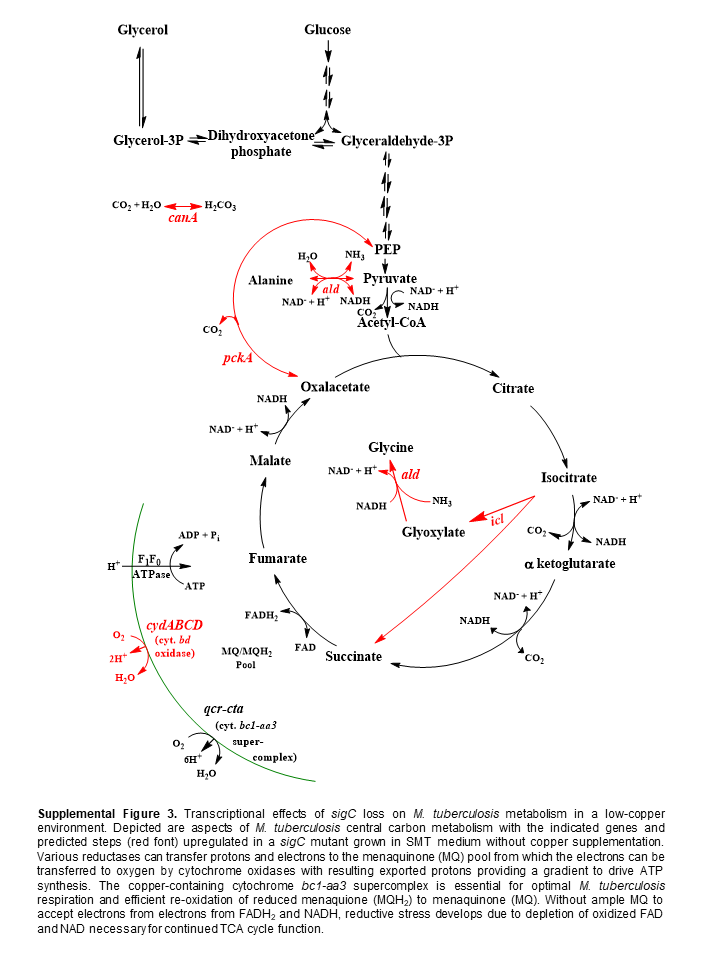

Supplement: Supplementary file 1 [file ijms-22-02118-s001.zip › Supplementary files/Figure S3.TIF]
